# Neferine ameliorates cardiomyoblast apoptosis induced by doxorubicin: possible role in modulating NADPH oxidase/ROS-mediated NFκB redox signaling cascade

**DOI:** 10.1038/s41598-017-12060-9

**Published:** 2017-09-25

**Authors:** Lohanathan Bharathi Priya, Rathinasamy Baskaran, Chih-Yang Huang, Viswanadha Vijaya Padma

**Affiliations:** 1 Translational Research Laboratory, Department of Biotechnology, School of Biotechnology and Genetic Engineering, Bharathiar University, Coimbatore, 641046 Tamil Nadu India; 2 National Institute of Cancer Research, National Health Research Institutes, Zhunan, Miaoli County Taiwan; 3 Graduate Institute of Basic Medical Science, China Medical University, Taichung, Taiwan; 4 Graduate Institute of Chinese Medical Science, China Medical University, Taichung, Taiwan; 5 Department of Health and Nutrition Biotechnology, Asia University, Taichung, Taiwan

## Abstract

Doxorubicin (DOX) mediated cardiomyopathy is a major challenge in cancer chemotherapy. Redox-cycling of doxorubicin by flavoenzymes makes the heart more vulnerable to oxidative stress leading to cardiac dysfunction. The present study evaluates the role of neferine, a bisbenzylisoquinoline alkaloid, in curbing the molecular consequences of DOX-exposure in H9c2 cardiomyoblasts. Neferine pre-treatment increased cell viability upon DOX-exposure. DOX activates NADPH oxidase subunits, (p22phox, p47phox, gp91phox) as the primary event followed by peak in [Ca^2+^]i accumulation by 2 h, ROS by 3 h and activated ERK1/2 and p38 MAPKinases, time dependently along with the activation and translocation of NFκB and up-regulated COX2 and TNF-α expressions. Neferine pre-treatment modulated NADPH oxidase/ROS system, inhibited MAPKinases and NFκB activation, reduced sub G1 cell population and concomitantly increased cyclin D1 expression reducing DOX-mediated apoptosis. The study demonstrates for the first time, the molecular sequential events behind DOX toxicity and the mechanism of protection offered by neferine with specific relevance to NADPH oxidase system, MAPKinases, inflammation and apoptosis in H9c2 cells. Our data suggests the use of neferine as a new approach in pharmacological interventions against cardiovascular disorders as secondary complications.

## Introduction

Anthracycline antineoplastic drug, doxorubicin (DOX) is a widely used chemotherapeutic agent in the treatment of different types of cancer including solid tumors, leukemias, lymphomas and breast cancer^1^. Despite being a potential chemotherapeutic agent, DOX usage is limited by side effects like immune suppression, vomiting, alopecia, extravasation and the most important cardiotoxicity^2^. Cardiotoxicity of DOX is mediated through dilated cardiomyopathy and congestive heart failure^3^. DOX exerts anticancer properties by DNA topoisomerase II; whereas DOX-induced cardiotoxicity is mediated through Reactive Oxygen Species (ROS), which leads to oxidative stress and apoptotic cell death^4^.

Mechanism of DOX-meditated ROS generation has not been fully understood so far. DOX is metabolized by purine nucleotide flavoproteins to an intermediate quinone which enters one-electron redox cycling resulting in the generation of superoxide ions (O_2_^●−^) and hydrogen peroxide (H_2_O_2_). Further H_2_O_2_ was decomposed into highly reactive ^●^OH by low molecular weight irons. Formation of DOX-iron complex also triggers ROS generation^5^. NADPH dehydrogenase and NADPH cytochrome P450 enzymes in mitochondria and sarcoplasmic reticulum are the primary target of DOX reduction to semiquinone and causes ultra-structural damage to these organelles^6^. NADPH oxidase complex present in the mitochondrial membrane is the potent source for the generation of ROS. NADPH acts as an electron donor to oxygen and generate O_2_^●−^ inside the mitochondria^7^. NADPH oxidase complex comprises a membrane-bound heterodimer consisting of a catalytic NOX2 (gp91phox) and p22phox subunits to which several cytosolic subunits such as p47phox, p67phox, p40phox, and Rac gets associated in the activated enzyme^8^. In cardiomyocytes and endothelial cells, ROS generated through NADPH oxidase has been reported to interfere with redox signaling^9^. Loss of NOX2 was reported to prevent oxidative stress and progression to advanced heart failure^8^. Hypergeneration of ROS by DOX leads to the oxidative stress, which in turn provokes apoptotic signaling cascade in cardiomyocytes^10^. DOX activates pro-inflammatory gene, Nuclear Factor-κB (NFκB) through ROS and toll-like receptor 2 (TLR2) mediated signaling, leading to cytokine production and apoptosis, which finally results in cardiac dysfunction^11^. Although there are compounds reported for preventing DOX-induced cardiac dysfunction, they have certain limitations^2^. Thus finding an ideal candidate with multiple actions for alleviating DOX-induced cardiotoxicity by modulating NADPH oxidase, ROS generation and apoptosis is important.

Neferine, a bisbenzylisoquinoline alkaloid present in the seed embryo of lotus (*Nelumbo nucifera* Gaertner) plumules has been reported to possess various physiological and pharmacological activities like anti-diabetic^12^, cholinesterase inhibitory^13^, anti-thrombotic^14^, sedative^15^ and anti-cancerous effects^16^. Our previous studies showed the sensitizing effect of neferine to low dose DOX in lung cancer cell line model^17^. Recently, we reported the anti-apoptotic potential of neferine against hypoxic challenge *in vitro* ^18,19^. However, the precise molecular mechanism of action of neferine on DOX-induced apoptosis has not been reported so far.

Based on the available data, the present study was designed to elucidate the modulatory role of neferine on NADPH oxidase mediated redox signaling through MAPKinases and NFκB pathway in DOX-induced toxicity in H9c2 cells.

## Results

### Neferine attenuates DOX-induced H9c2 cell death

**Figure 1:**
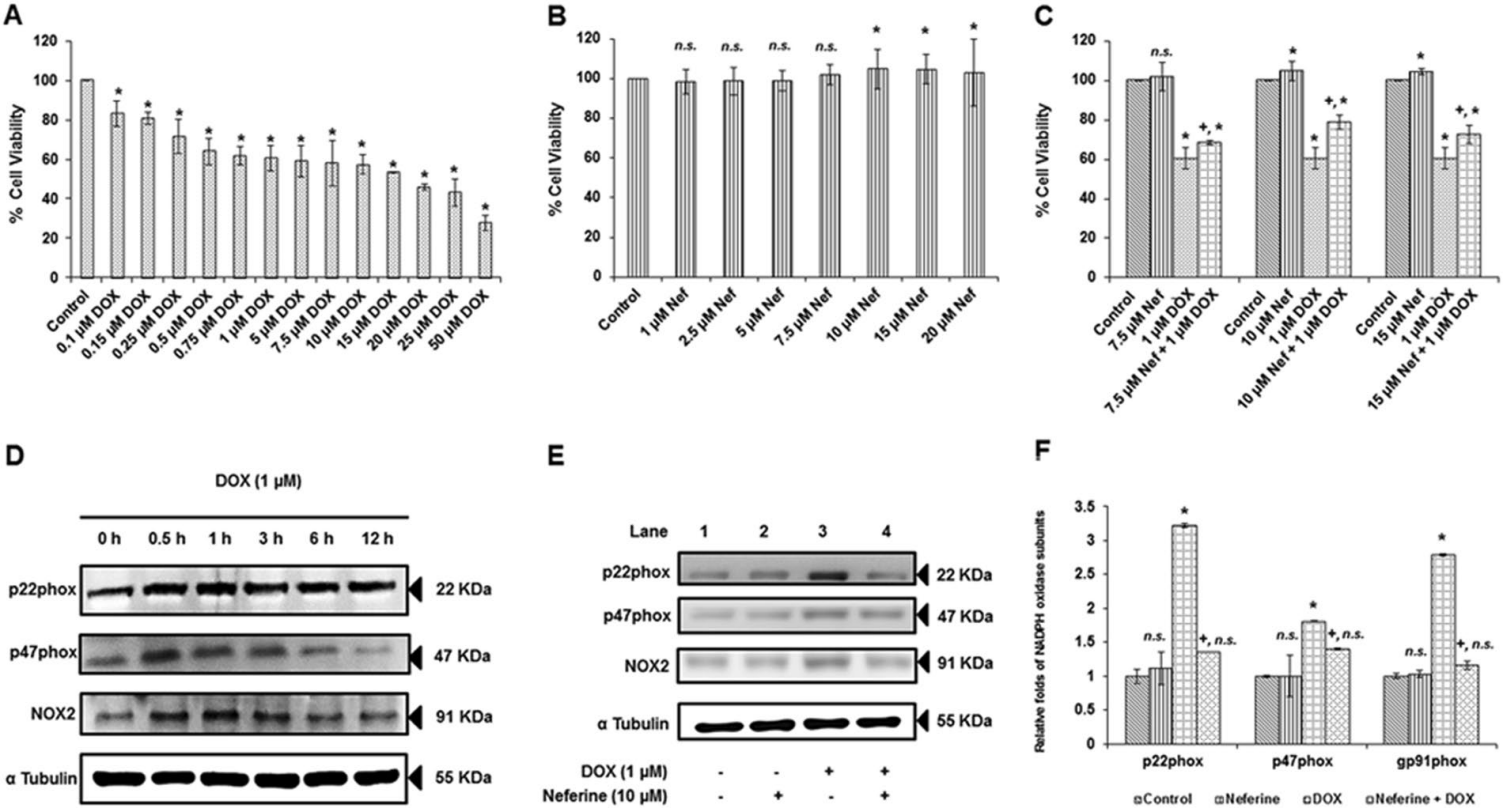
Effect of neferine and DOX-treatment on H9c2 cell viability and NADPH oxidase subunits expression (**A**) Effect of DOX on H9c2 cell viability for 24 h. (**B**) Effect of neferine on H9c2 cell viability for 24 h. (**C**) H9c2 cells were pre-treated with 7.5, 10 and 15 µM of neferine for 24 h followed by treatment with 1 µM DOX for 24 h and analyzed for cell viability by MTT cell proliferation assay. (**D**) DOX-induced NADPH oxidase subunits in a time dependent manner in H9c2 cells (0 h, 0.5, 1 h, 3 h, 6 h and 12 h). (**E**) Neferine pre-treatment down-regulates DOX-induced NADPH oxidase subunits-gp91phox, p22phox and p47phox expression in H9c2 cardiomyoblast cells. (**F**) Densitometry analysis of the protein bands of NADPH oxidase subunits. The results shown are mean ± s.d. of six individual experiments performed in triplicates for cell viability experiments and mean ± s.d. of three individual experiments for the expression analysis of NADPH oxidase subunits. ^*^p < 0.05 significantly different from control, ^+^p < 0.05 significantly different from DOX treated cells, n.s. = non-significantly different from control (one way ANOVA followed by Tukey’s multiple comparison).

DOX-treatment, between 0.1 and 50 µM for 24 h resulted in a dose dependent H9c2 cell death (Fig. 1A). 1 µM DOX-treatment reduced the cell viability to 60%. Also, this particular concentration was clinically relevant to the dose of DOX present in plasma of patient undergoing DOX- chemotherapy^20^. Hence, further experiments were carried with 1 µM DOX. H9c2 cells incubated with neferine alone (between 1 and 5 µM) for 24 h offered maintenance in cell viability similar to control group. Treatment with 7.5, 10 and 15 µM neferine alone showed significant proliferative effect compared to control (Fig. 1B). Pre-treatment with 7.5, 10 and 15 µM neferine followed by DOX-treatment for 24 h offered significant increase in cell viability (78%) at 10 µM neferine pre-treatment compared to 7.5 (68% cell viability) and 15 µM (72% cell viability) neferine pre-treatment (Fig. 1C). Based on these results, 10 µM neferine with better protective effect was chosen for further studies.

### Neferine down-regulates DOX-induced gp91phox (NOX2), p22phox and p47phox expression in H9c2 cells

NADPH oxidase system comprise of defined subunits as membrane bound and cytosolic components. NOX2, p22phox and p47phox are the important subunits in the NADPH oxidase system responsible for the stability and activity of the complex. Hence expression pattern of these subunits was analyzed in a time-course study (0 h, 0.5, 1 h, 3 h, 6 h and 12 h). DOX-treatment in the H9c2 cells markedly induced the expression of NOX2, p22phox, and p47phox in a time dependent manner. We found that protein expression of p22phox, p47phox and gp91phox starts after immediate exposure of DOX from 1 h (Fig. 1D). From these results, it was apparent that DOX-induced NOX system was the primary event in DOX-induced cardiotoxicity. H9c2 cells pre-treated with neferine (10 µM) for 24 h followed by DOX (1 µM) for 24 h revealed a significant suppression in the expression of these subunits (Fig. 1E,F).

### Neferine inhibited the ROS hypergeneration by DOX in H9c2 cells

**Figure 2:**
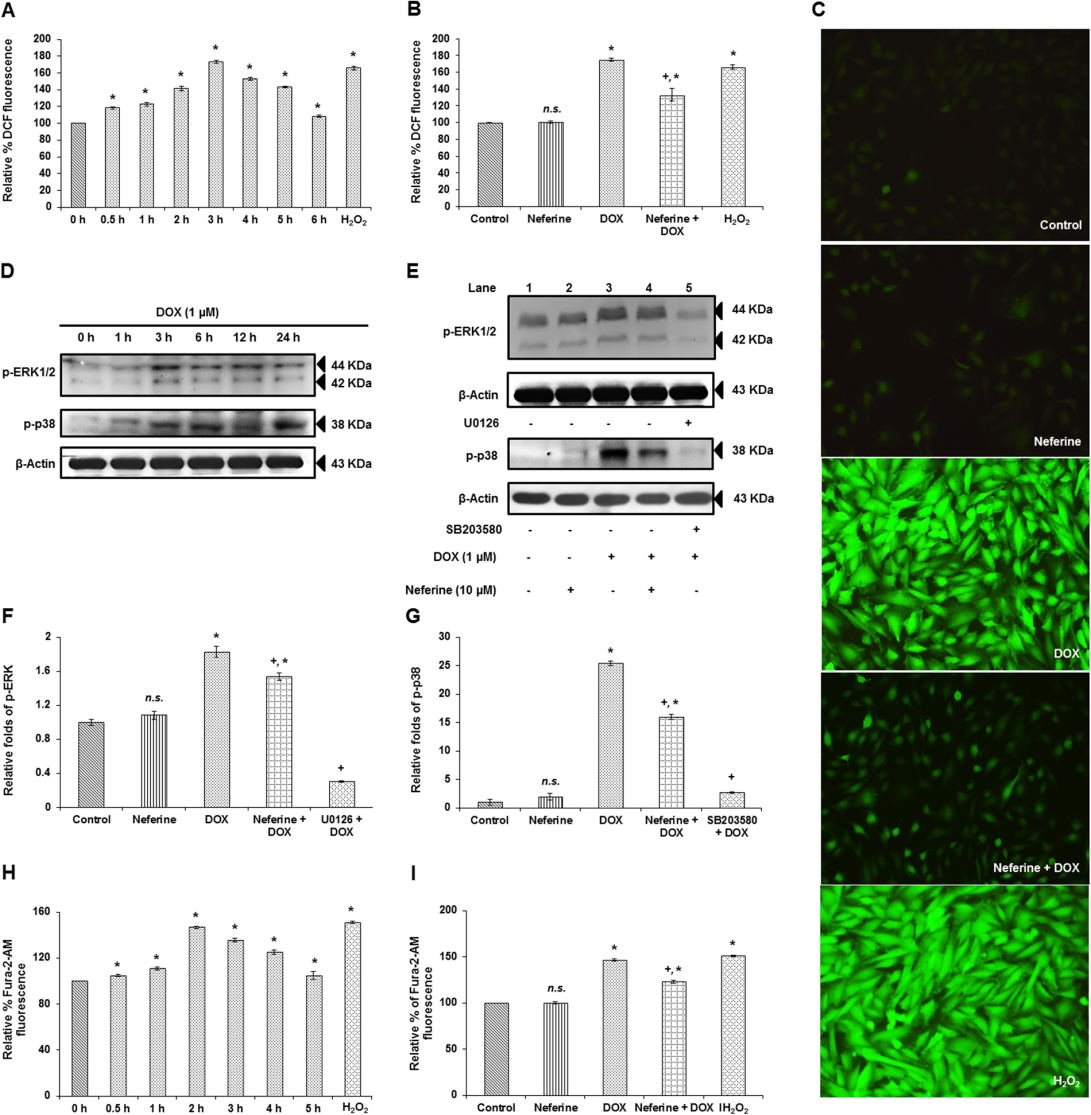
Effect of neferine and DOX on cellular ROS, ERK1/2 and p38 activation and intracellular calcium levels (**A**) ROS levels in H9c2 cells exposed to varying time points of DOX (0 h, 0.5 h, 1 h, 2 h, 3 h, 4 h, 5 h and 6 h). (**B**) Effect of neferine pre-treatment for 24 h followed by DOX-exposure on ROS levels at 3 h time point. (**C**) Microscopic analysis of neferine and DOX-treatment on ROS levels by DCF Fluorescence. (**D**) Time dependent phosphorylation levels of MAPKinases, ERK1/2 and p38 (0 h, 1 h, 3 h, 6 h, 12 h and 24 h). (**E**) Neferine pre-treatment significantly reduced the activation of the MAPKinases, ERK1/2 and p38 induced by DOX-treatment in H9c2 cells. The results were confirmed in the presence of ERK1/2 (U0126) and p38 (SB203580) inhibitors. (**F,G**) Densitometry analysis of the protein bands of p-ERK1/2 and p-p38. (**H**) Effect of DOX-treatment alone on [Ca^2+^]i levels at different time points (0 h, 0.5 h, 1 h, 2 h, 3 h, 4 h and 5 h). (**I**) Effect of neferine pre-treatment on [Ca^2+^]i levels followed by DOX-exposure. Cells treated with 100 µM H_2_O_2_ for 30 min served as positive control. The graphs shown are mean ± s.d. of six individual experiments performed in triplicates for determination of cellular ROS and intracellular calcium accumulation and mean ± s.d. of three individual experiments for the analysis of ERK1/2 and p38 activation. ^*^p < 0.05 significantly different from control, ^+^p < 0.05 significantly different from DOX treated cells, n.s. = non-significantly different from control (one way ANOVA followed by Tukey’s multiple comparison).

ROS plays a vital role in developing cardiac dysfunction under various pathological conditions. Since, DOX-potentiated the NADPH oxidase system (a major source for cellular ROS generation), we measured the ROS generation in H9c2 cells in a time course experiment (0 h, 0.5 h, 1 h, 2 h, 3 h, 4 h, 5 h and 6 h) to determine the peak in generation of DOX-induced ROS. DOX-induced ROS generation started from 0.5 h, peaked at 3 h and declined after 3 h time point (Fig. 2A). Neferine pre-treatment significantly reduced the ROS generated due to DOX-exposure. No significant change was observed in the group which received neferine treatment alone (Fig. 2B). The same results were confirmed using fluorescent imaging, where DOX-treatment showed higher fluorescent intensity of DCF when compared to control. Neferine pre-treatment followed by DOX-treatment showed a marked reduction in DCF fluorescence when compared to DOX-alone treated group (Fig. 2C).

### Neferine pre-treatment suppressed DOX-induced activation of ERK1/2 and p38 MAPKinases

MAPKinases are known to get activated in response to ROS. H9c2 cells were treated with DOX for various time points (0 h, 1 h, 3 h, 6 h, 12 h and 24 h) and analyzed for the activation of MAPKinases, ERK1/2 and p38. Activation of these proteins was significant in all the time points compared to control. Peak in expression and activation of ERK1/2 was observed at 3 h of DOX-exposure compared to other time points, whereas the peak in activation and expression levels of p38 was observed at 24 h time point of DOX-treatment (Fig. 2D). Neferine pre-treatment effectively modulated DOX-induced ERK1/2 and p38 MAPKinase activation, evident with the reduced phosphorylation levels of p-ERK1/2 and p-p38. The results were confirmed by exposing the DOX-treated H9c2 cells to SB203580 (p38 inhibitor), which resulted in a decreased phosphorylation of p38 compared to DOX-alone treated group (Fig. 2E–G).

### Neferine pre-treatment reduced intracellular calcium [Ca^2+^]i accumulation in H9c2 cells

ROS and H_2_O_2_ generated by DOX disturb the function of sarcoplasmic reticulum and alter calcium (Ca^2+^) homeostasis. Ca^2+^ accumulation in the cardiomyocytes plays an important role in the pathogenesis of DOX-induced cardiomyopathy^2^. Measurement of [Ca^2+^]i levels using Fura 2/AM in H9c2 cells at different time points (0 h, 0.5 h, 1 h, 2 h, 3 h, 4 h, and 5 h) showed a peak in accumulation by 2 h of DOX-exposure significantly different from control (Fig. 2H). Whereas pre-treatment with neferine for 24 h markedly reduced the Ca^2+^ accumulation inside the H9c2 cells (Fig. 2I).

### DOX-induced nuclear translocation of NFκB, expression of COX2 and TNF-α was attenuated by neferine treatment

**Figure 3:**
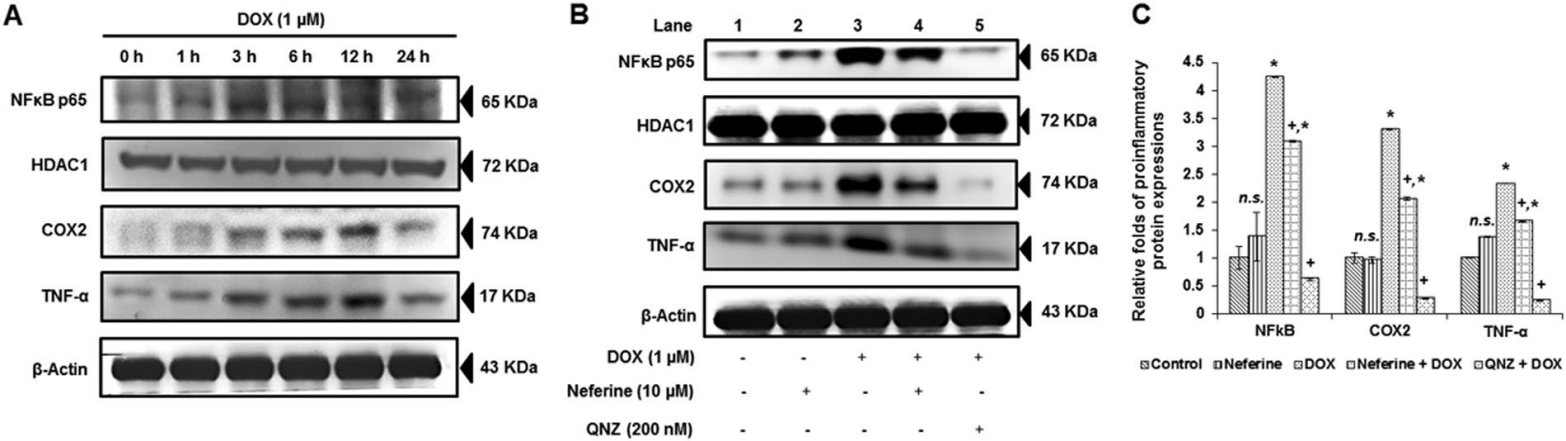
Effect of neferine and DOX on the pro-inflammatory transcription factor, NFκB p65 and its downstream regulators, COX2 and TNF-α (**A**) Time dependent expression patterns of NFκB (nuclear extract) and COX2 and TNF-α (cytoplasmic extract) (0 h, 1 h, 3 h, 6 h, 12 h and 24 h). (**B**) DOX-induced nuclear translocation of NFκB, expression of COX2 and TNF-α was reduced by neferine treatment. Results were confirmed in the presence of NFκB inhibitor (QNZ). (**C**) Densitometry analysis of the protein bands of NFκB, COX2 and TNF-α. The results shown are mean ± s.d. of three individual experiments. ^*^p < 0.05 significantly different from control, ^+^p < 0.05 significantly different from DOX treated cells, n.s. = non-significantly different from control (one way ANOVA followed by Tukey’s multiple comparison).

NFκB is a crucial pro-inflammatory transcriptional factor activated in response to various stimuli including ROS. Activation and nuclear translocation of NFκB p65 may trigger inflammatory response and apoptosis^18^. Expression analysis of NFκB p65 in the nuclear extract revealed a significant increase upon DOX-exposure at various time points. A peak in expression levels of NFκB p65 was found at 3 h of DOX-treatment when compared with other time points of DOX-treatment. Analysis on the downstream regulators of NFκB p65, such as COX2 and TNF-α also showed a time dependent response to DOX-treatment (Fig. 3A). Neferine pre-treatment for 24 h followed by DOX-exposure for 3 h reduced the levels of NFκB p65 in the nuclear extract with a subsequent suppression in the expression levels of COX2 and TNF-α upon DOX-treatment. The results were confirmed by exposing the DOX-treated H9c2 cells to QNZ (NFκB p65 inhibitor), which resulted in inhibition of NFκB p65 nuclear translocation and suppression of the downstream targets, COX2 and TNF-α expressions compared to DOX-alone treated group (Fig. 3B,C).

### Neferine pre-treatment improves mitochondrial membrane potential in DOX-treated cells

**Figure 4:**
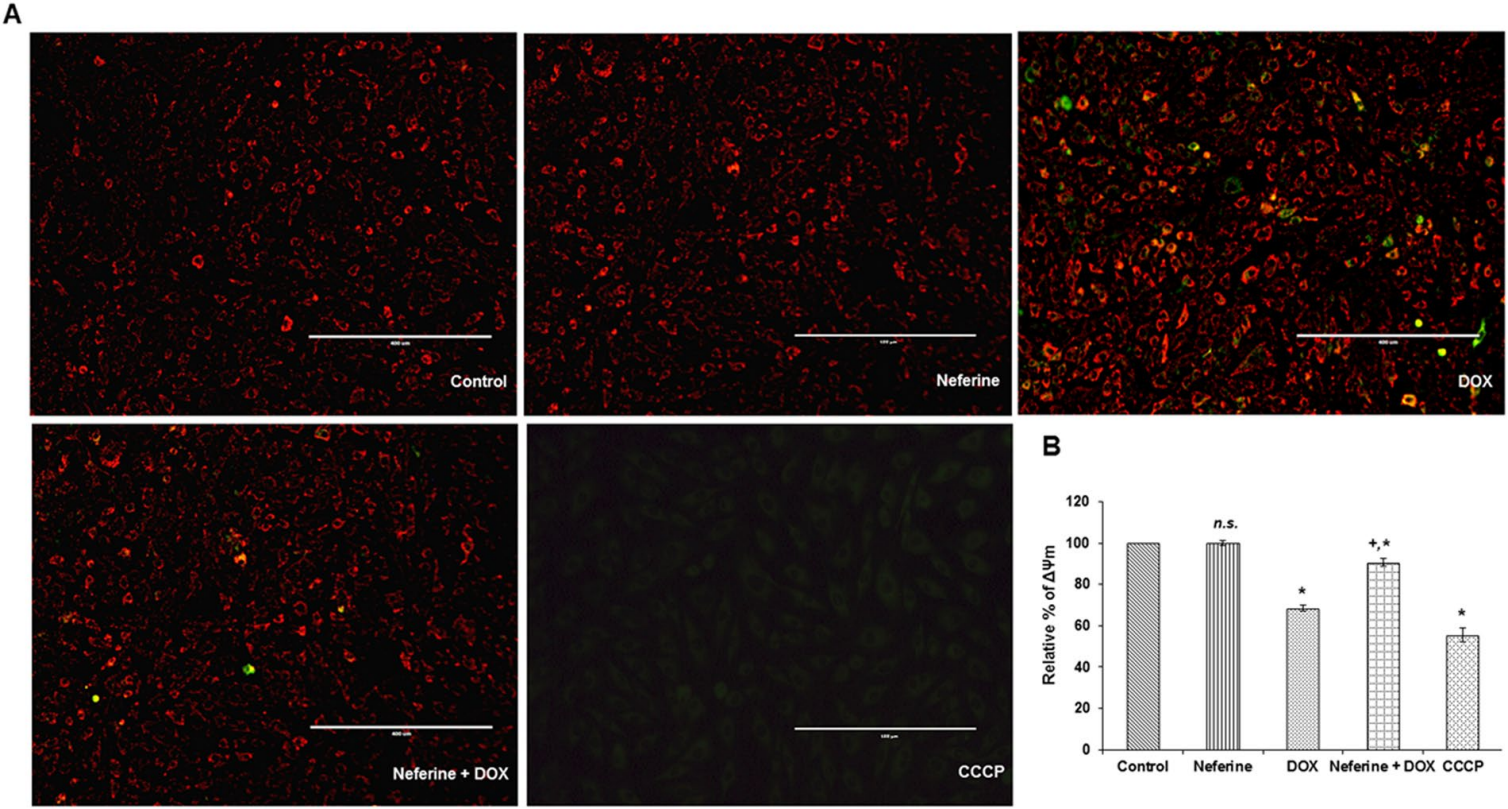
Effect of neferine and DOX on mitochondrial membrane potential in H9c2 cells (**A**) Microscopic analysis of the mitochondrial membrane potential upon neferine and DOX-treatment using JC-1 fluorescent dye. (**B**) Effect of neferine pre-treatment on the dissipation in ΔΨm by DOX using DiOC6 fluorescent dye. Graph represents mean ± s.d. of six individual experiments performed in triplicates. ^*^p < 0.05 significantly different from control, ^+^p < 0.05 significantly different from DOX treated cells, n.s. = non-significantly different from control (one way ANOVA followed by Tukey’s multiple comparison).

Maintenance of mitochondrial membrane potential (ΔΨm) is vital for cell survival. Dissipation in ΔΨm may lead to apoptosis^18^. DOX-exposure to H9c2 cells resulted in a significant loss of ΔΨm compared to control. CCCP, a mitochondrial uncoupler also caused ΔΨm loss in the cardiomyoblast. A significant increase in ΔΨm was observed in cells which received neferine pre-treatment followed by DOX-exposure, compared to DOX-alone treated cells. Neferine alone pre-treated cells showed no significant effect on ΔΨm (Fig. 4A,B).

### Neferine treatment limits increase in sub G1 apoptosis induced by DOX-treatment in H9c2 cells

**Figure 5:**
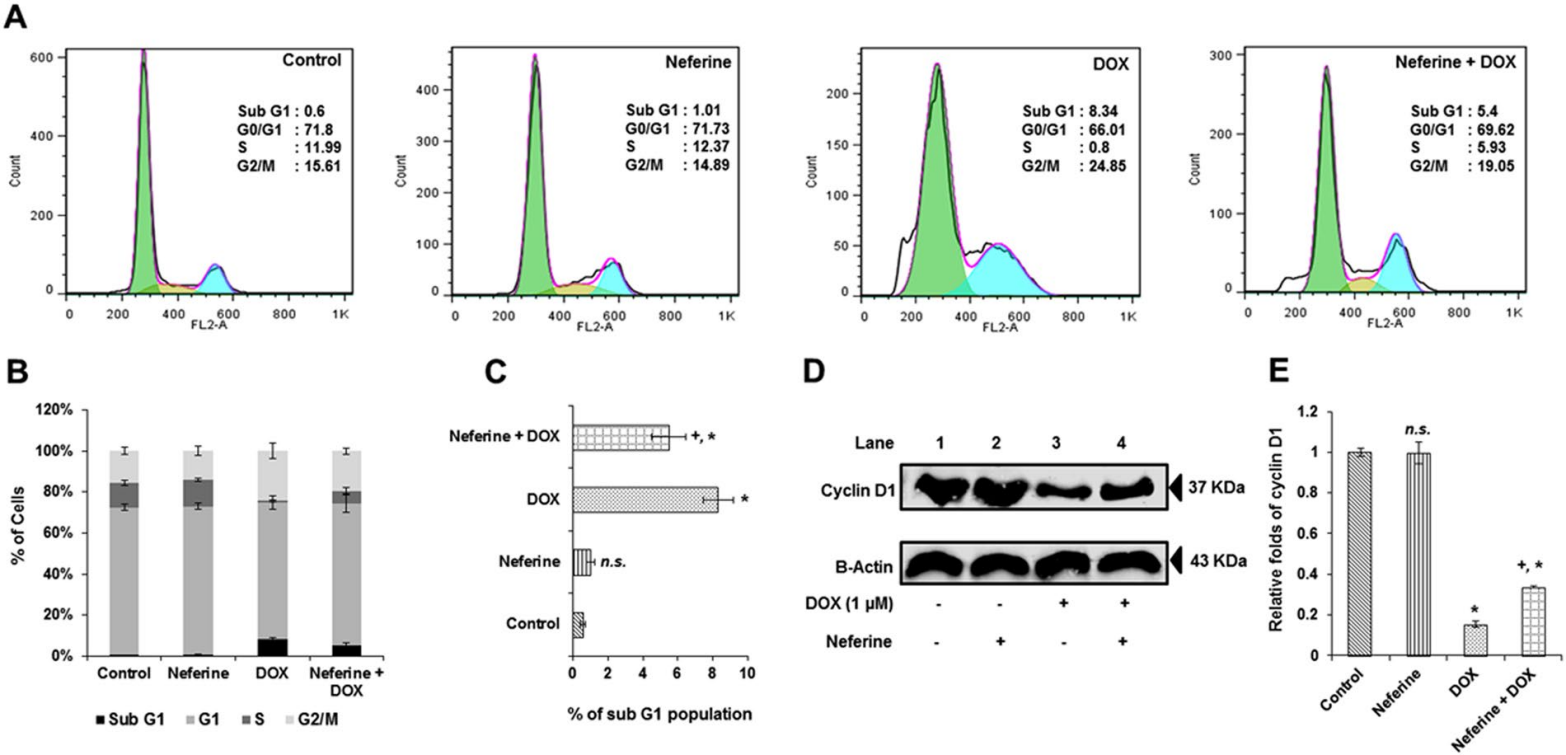
Effect of neferine and DOX on cell cycle regulation (**A**) Flow cytometry analysis of cells upon neferine and DOX treatment. (**B**) % accumulation of H9c2 cells at sub G1, G0/G1, S and G2/M phase in different treatment groups. (**C**) Effect of neferine and DOX on sub G1 apoptosis. **(D)** Effect of neferine and DOX on the cell cycle regulatory protein, cyclin D1 expression levels. (**E**) Densitometry analysis of the protein bands of cyclin D1. Results shown are mean ± s.d. of three individual experiments performed in triplicates and mean ± s.d. of three individual experiments for the analysis of cyclin D1 expression. ^*^p < 0.05 significantly different from control, ^+^p < 0.05 significantly different from DOX treated cells, n.s. = non-significantly different from control (one way ANOVA followed by Tukey’s multiple comparison).

DOX (1 µM) treatment lead to a significant increase in sub G1 apoptosis (8.3%) compared to control cells (0.5%). Whereas H9c2 cells pre-treated with neferine significantly reduced the DOX-induced sub G1 apoptosis to 5.4% (Fig. 5A–C). In order to confirm the above results, expression levels of cell cycle protein, cyclin D1 was measured using western blot analysis. DOX-reduced the expression levels of cyclin D1 when compared to control. Neferine pre-treatment significantly increased the expression levels of cyclin D1 in H9c2 cells compared to DOX-alone treated cells (Fig. 5D,E).

### Neferine significantly reduces DOX-induced DNA breaks, intrinsic apoptosis and morphological changes in H9c2 cardiomyoblast cells

**Figure 6:**
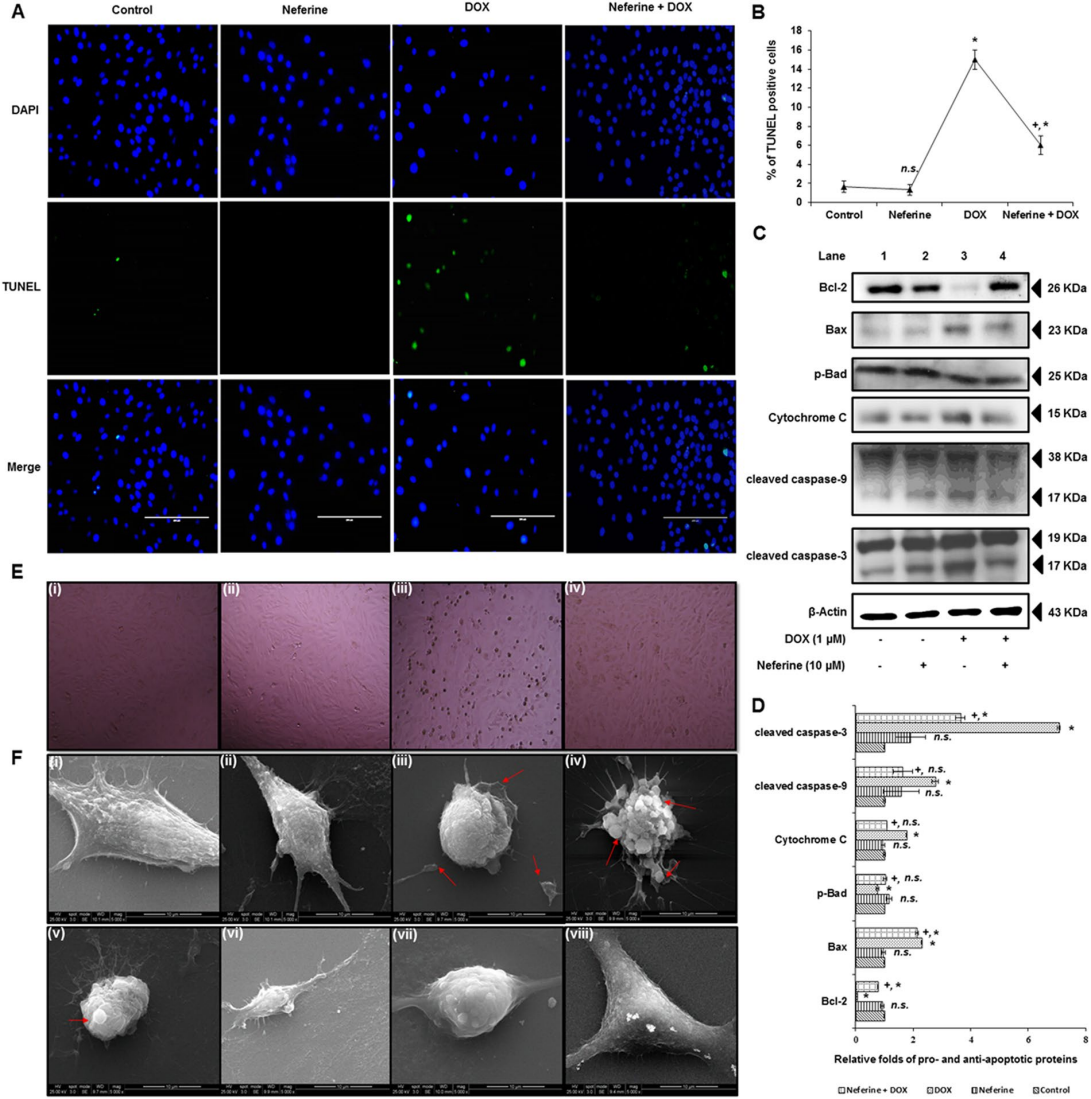
Effect of neferine and DOX-treatment on DNA breaks, intrinsic apoptosis and morphological changes in H9c2 cells (**A**) Terminal deoxynucleotidyl transferase (TdT) dUTP nick-end labeling showing reduced DNA breaks and apoptotic body formation by neferine pre-treatment. **(B)** % of TUNEL positive apoptotic nuclei. **(C)** Western blot analysis of neferine and DOX-treatment on pro- and anti-apoptotic protein levels. (**D**) Densitometry analysis of the protein bands of pro- and anti-apoptotic proteins. (**E**) Phase-contrast microscopic images of H9c2 cells. (i) Control cells. (ii) Neferine treated cells showing normal morphology. (iii) DOX-alone treated cells showing spindle shape shrinkage and detachment of cells from culture surface. (iv) Cells pre-treated with neferine followed by DOX-treatment reduced the shrinkage and rounding up of cells from the culture surface. (**F**) Morphological analysis of H9c2 cells by scanning electron microscopy. (i & ii) Control and neferine treatment showing the normal morphological architectures of elongated spindle shaped H9c2 cells with dense surface. (iii-v) DOX-treatment showing morphological changes like loss of spindle shape, cell shrinkage, rounding up of cells and membrane blebbing associated with apoptosis in H9c2 cells. (vi-viii) Neferine pre-treatment showing reduced structural abnormalities induced by DOX- treatment. The graphs represent mean ± s.d. of three individual experiments. ^*^p < 0.05 significantly different from control, ^+^p < 0.05 significantly different from DOX treated cells, n.s. = non-significantly different from control (one way ANOVA followed by Tukey’s multiple comparison).

Results of TUNEL assay confirmed the apoptotic event in DOX-treated H9c2 cells, which is evident from the formation of DNA breaks and apoptotic body formation (Fig. 6A,B) with a significantly decreased expression of the anti-apoptotic protein, Bcl-2 and phosphorylated Bad. This happened with simultaneous induction of cytochrome c release, increased Bax expression, activated caspase-3, caspase-9 and PARP cleavage significantly upon DOX-exposure to H9c2 cells, compared to control (Fig. 6C,D). Analysis on the structural architecture of H9c2 cells by phase contrast microscopy showed spindle shape shrinkage and detachment of cells from culture surface after DOX-treatment (Fig. 6E). Analysis of morphology by scanning electron microscopy showed the normal morphological architectures of elongated spindle shaped H9c2 cells with dense surface in control and neferine alone treated cells. DOX-treatment induced morphological changes like loss of spindle shape, cell shrinkage, rounding up of cells and membrane blebbing associated with apoptosis in H9c2 cells. Neferine pre-treatment effectively reduced DOX-induced DNA breaks and alterations in the expressions of pro- and anti-apoptotic proteins and reduced the structural abnormalities in H9c2 cells (Fig. 6A–F).

## Discussion

ROS generated via NADPH oxidase system is a major contributor to the cardiac dysfunction or cardiomyopathy induced by DOX^6^. NOX2 and NOX4 isoforms of NOX family, associated with p22phox are predominantly expressed in cardiomyocytes^21^. NOX2 also referred to as gp91phox, present in sarcolemma forms a transmembrane heterodimer complex by post-translational modification with other regulatory subunits (p47phox, p67phox, p40phox and Rac1) and p22phox upon activation by agonist. Active NOX2-p22phox complex plays a crucial role in cardiac redox signaling^22^. NOX2 plays a central role in heart failure by activation of MAPKinases and NFκB through stimuli by mechanical stress, angiotensin II and endothelin-1 in myocardium^21^. Pharmacological inhibition of NOX system by apocyanin or genetic deletions of NOX2 or p47phox subunit have shown to possess cardioprotective effect by inhibition of ROS production^23–25^. Herein we discussed the time dependent expressions of the key components of NADPH oxidase system, ROS generation, MAPKinases phosphorylation and nuclear translocation of NFκB in a sequential manner which finally resulted in myocardial apoptosis upon DOX-exposure and the counteracting effect by neferine pre-treatment.

Results of our present study showed increased expressions of NOX2/gp91phox, p47phox and p22phox in a time dependent manner in H9c2 cardiomyoblast as the primary event triggered in response to DOX. Brandt, *et al*.^26^ observed the activation of NOX2, p22phox in addition to Rac1 subunit expression in membrane fractions of cultured cardiomyocytes as well as in alcoholic cardiomyopathy animal models^26^. The induction of NOX subunits may be due to the metabolic activation of DOX by NOX system of H9c2 cells. Cheng *et al*. have reported the impairment in ubiquitination and proteosomal degradation of NOX subunits such as gp91phox, p47phox and p22phox in DOX-mediated cardiotoxicity^27^. The ROS quenching efficacy of neferine by its antioxidant property might have suppressed the induction in the expression levels of these subunits.

Alterations in calcium transport inside the myocardial cells have a close association with myocardial remodeling. Excess Ca^2+^ accumulation by calcium transporters might lead to decreased cardiac systolic potential and increased frequency of sudden cardiac death^28^. Intracellular Ca^2+^ accumulation peaked at 2 hr after exposure to DOX in our study. DOX-mediated Ca^2+^ overload in cardiac cells results in mitochondrial calcium overload and alterations of energy metabolism which in turn promote ROS generation^29^. ROS-associated opening of ryanodine receptor (RyR) might trigger the release of Ca^2+^ ions from sarcoplasmic reticulum in the myocardium^30^. NOX2 derived ROS sensitize RyR and enhance Ca^2+^ release in cardiomyocytes. Also, the expression levels/open-state probability of L-type calcium channels may be modulated by angiotensisn-II/endothelin I via NOX2^21^. Elevated Ca^2+^ concentration in myocardium have been shown to trigger caspase-12, which results in cardiac dysfunction^31^. Neferine prolonged action potential duration by blocking L-type calcium channel in *in vitro* and *in vivo* arrhythmic models^29^. Neferine inhibits [Ca^2+^]i induced by ADP and prevents the internal release of Ca^2+^ ^32^. Reduction in the levels of DOX-mediated ROS by neferine was evident from our study. Preventing ROS mediated RyR opening might be a crucial factor in reducing DOX-induced cardiotoxicity.

ROS generation in response to DOX-initiated at 30 min and peaked at 3 h and lead to dissipation of ΔΨm in the present study. Several reports have shown that NOX and mitochondrial electron transport chain are the major source for ROS generation in cardiac cells^33,34^. NADPH oxidase activated by angiotensin II triggers O_2_^●−^ production in mitochondria, which confirms the cross talk between NADPH oxidase system and mitochondrial ROS^35^. Elevation in the total cellular ROS upon DOX-treatment in the present study might have also been from the crosstalk between mitochondrial ROS and NADPH oxidase system in H9c2 cardiomyoblasts. Activation of NADPH oxidase produces superoxide via angiotensin II receptor type 1, leads to activation of kinases (protein kinase C (PKC) and Src-kinase). PKCε, a redox sensitive kinase in mitochondria was activated by superoxide produced by NADPH oxidase^35^. Hyper generation of ROS initiates the opening of mitochondrial permeability transition pores (MPTP) and release free radicals across inner and outer mitochondrial membrane^36^, which results in decreased mitochondrial membrane potential. Persistence of MPTP opening for a longer duration causes swelling of mitochondria and myocardial damage^37^. Reduction in the levels of ROS generation and dissipation of ΔΨm might be due to the free radical scavenging and Ca^2+^ channel blocking activity of neferine. Similar results were reported earlier by Dong *et al*. as well as Qian *et al*.^32,38^.

ROS acts as a stimuli and modulates several signaling kinases including MAPKinases, PI3K/Akt, protein kinase C and Wnt/β-catenin^39^. NOX mediated ROS hypergeneration in myocardial cells activates MAPKinases such as ERK1/2 and JNK, which may lead to TNF-α production and myocardial dysfunction^40^. DOX activates JNK/ERK through NADPH oxidase dependent mechanism while p38 expression is mediated through NADPH oxidase independent mechanism in cardiomyocytes^41^. Time course elucidation on the molecular downstream regulators of ROS generated by DOX showed a time dependent activation of ERK1/2 and p38 MAPKinases. Neferine pre-treatment reduced the activation by phosphorylation of ERK1/2 and p38 kinases caused by DOX-exposure. Neferine exhibited anti-fibrogenic effect by inhibiting the activation of ERK1/2 and p38 MAPKinases signaling in cardiac fibroblasts^42^.

Redox-sensitive activation of signaling kinases like ERK, p38 and JNK via NOX dependent or independent manner leads to the induction of transcription factors like NFκB, HIF1-α (hypoxia inducible factor-1a) and Nrf2, which may prevent apoptosis or cardiac fibrosis in causing myocardial damage^43^. NFκB, a pro-inflammatory transcription factor plays a crucial role in deciding the cell fate. Activation and nuclear translocation of NFκB could trigger apoptosis and inflammation in heart^44^. Under normal physiological conditions, NFκB remains in an inactive form in cytoplasm by association with IκB. Upon stimuli by ROS or other kinase signaling, IκB dissociates and undergo ubiquitination and proteasomal degradation, then frees NFκB to translocate into nucleus which acts as a transcription activator for the genes involved in inflammation^45^. COX2, one of downstream regulator of NFκB, was known to be involved in inflammation. Preventing inflammatory responses in heart using interleukin converting enzyme inhibitors and anti-TNF-α monoclonal antibody improves cardiac function against diabetes induced cardiomyopathy^46^. DOX-induced inflammatory response occurred through NFκB pathway was confirmed by the use of NFκB specific inhibitor in the present study. The activation of pro-inflammatory genes NFκB, COX2 and TNF-α happened in a time dependent manner starting from 1 hr to 24 hr. H9c2 cells exposed to DOX for 3 h translocated NFκB to the nucleus and subsequently stimulated the expressions of COX2 and TNF-α. Neferine influenced the activation of NFκB pathway by reduced activation and translocation of NFκB to the nucleus during DOX-treatment. Neferine by virtue of its antioxidant property could have reduced the ROS/MAP kinase mediated nuclear translocation of NFκB, modulating the expression of its downstream regulators COX2 and TNF-α. Nuclear translocation of NFκB and up-regulation of COX2 and TNF-α was prevented by NFκB inhibitor QNZ in presence of DOX. Reduction in the nuclear translocation of NFκB by neferine pre-treatment was reported in our previous studies against hypoxic challenge in muscle cells^18^. Anti-amnesic effects of neferine by antioxidant and anti-inflammatory properties along with the inhibition of ChEs and BACE1 were reported^15^. Here we confirmed the anti-inflammatory role of neferine by modulating the redox sensitive activation of NFκB pathway against DOX-exposure.

NADPH oxidase mediated activation of MAPKinases may modulate variety of biological functions including gene expression, cell cycle control, cell survival and apoptosis. NADPH oxidase may also promote cell survival signaling pathway as cellular adaptation to stress or it may promote apoptosis in irreversibly damaged cells^47^. Apoptosis in cardiomyocytes reduced the myocardial cell population and leads to myocardial remodeling^48^. Increasing evidences demonstrate that, sustained myocardial apoptosis might lead to severe loss in myocardial function and heart failure^2,48,49^. Apoptotic cell death occurs during different pathological or physiological stimuli are controlled by a set of signaling proteins. Mitochondrial dependent intrinsic apoptosis was the main cause for myocardial loss and cardiac dysfunction in DOX-induced cardiotoxicity. Pro-apoptotic protein Bax, cytochrome c, caspase-9 and caspase-3 are up regulated during DOX-exposure, promoting myocardial apoptosis^50^ which is in corroboration with our present results with a significant down-regulation on the anti-apoptotic protein Bcl-2 along with decreased p-Bad levels. Also, DOX-treatment lead to a significantly increased % of sub G1 population by down regulating the cyclin D1, the central regulator of cell cycle and caused double strand DNA breaks, suggesting the key role of NADPH oxidase in DOX-mediated intrinsic pathway of apoptosis in H9c2 cardiomyoblasts. Activation of mitochondrial pathway of apoptosis might be due to the accumulation of [Ca^2+^]i and dissipation of ΔΨm upon DOX-exposure. DOX-induced oxidative stress and cardiac apoptosis through down-regulation of PI3K/Akt and activation of p38/JNK were suppressed by taurine treatment in rats^31^. Inhibition of NOX system by DPI/apocyanin reduced DOX-induced ROS production, caspase-3 activity and sub-G1 cell population^51^. Neferine with its anti-apoptotic property^18,52^ inhibits the cardiomyoblast apoptosis during DOX-exposure. The anti-apoptotic property of neferine may be attributed to the suppression of oxidative stress by its antioxidant nature and by the enhancement of endogenous enzymic antioxidant activity.

Changes in the microstructures of myocytes lead to altered myocardial function and myocardial loss. H9c2 cells displaying normal morphological architecture with elongated spindle shape helps for the vital electrophysiological function of myocardium^53^. Morphological alterations such as cell shrinkage, rounding up, membrane blebbing and translocation of phosphatidylserine (PS) from the inner to the outer leaflet of the plasma membrane are characteristic features of the cells undergoing apoptosis^54^ which ratifies our present results upon DOX-exposure. Neferine attenuated these structural abnormalities and maintained near normal cellular functions. Protection of the histoarchitecture of myocardium by neferine pre-treatment was shown against isoproterenol induced cardiotoxicity^19^.

**Figure 7:**
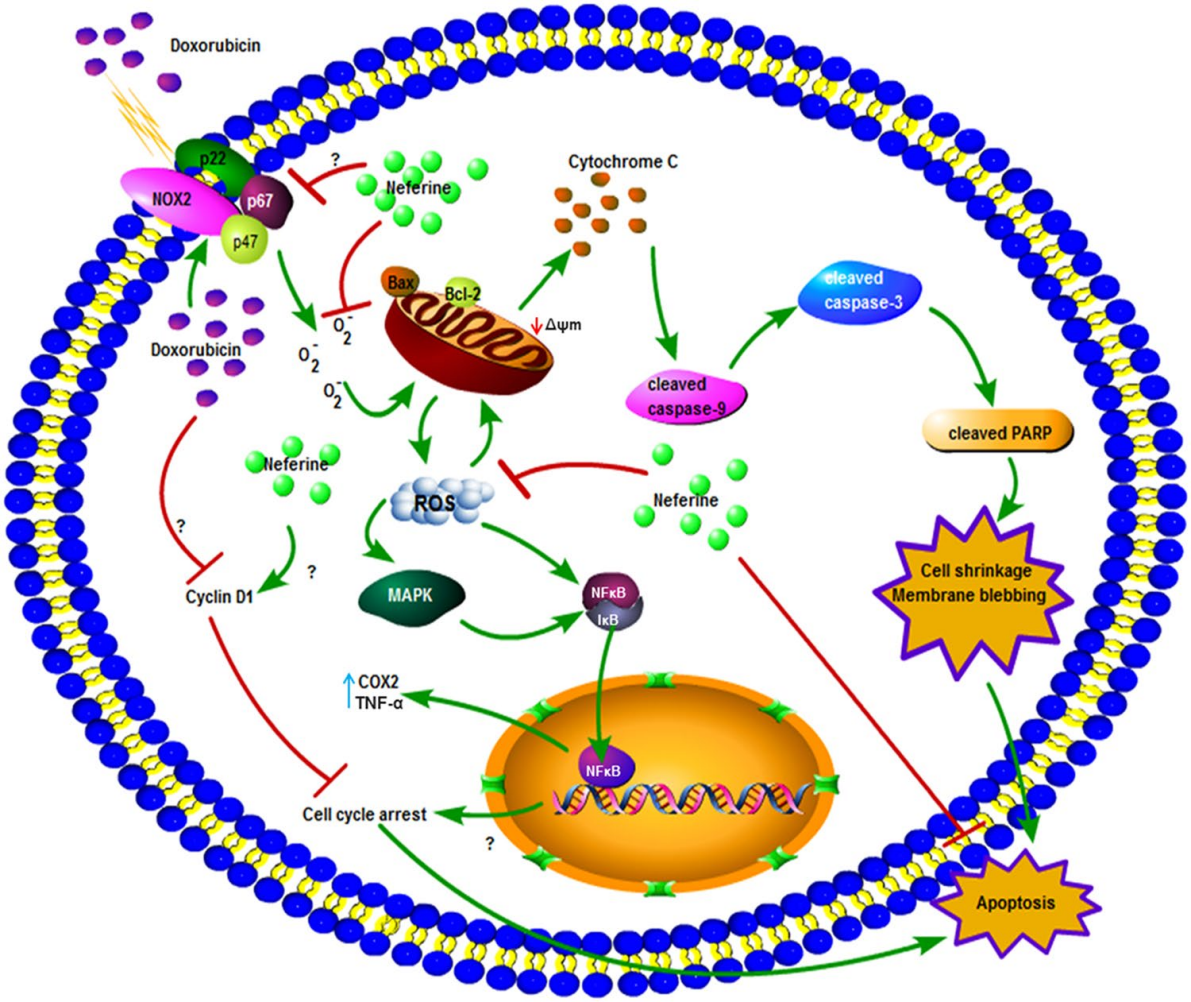
Schematic representation, behind the action of neferine on DOX-mediated apoptosis in H9c2 cardiomyoblasts.

Activation of NADPH oxidase and ROS hypergeneration disrupts the redox balance of H9c2 cardiomyoblasts, results in the activation of stress kinases (ERK1/2, p38) and the pro-inflammatory cytokines, NFκB, COX2 and TNF-α which finally leads to apoptotic cell death upon DOX-exposure. Neferine inhibited NFκB activation and decreased TNF-α, IL-6 and endothelin-1expressions in bleomycin induced pulmonary fibrosis^55^. Also, neferine attenuated hyperglycemia-induced endothelial cell apoptosis by suppressing ROS/Akt/NFκB signalling^52^. The maintenance in the structural integrity of H9c2 cells by neferine pre-treatment might be due to the antioxidant and anti-inflammatory nature of neferine curbing the converging effects of redox-signaling and activation of apoptotic cascade as an ultimate event in DOX-induced cardiotoxicity. The results of the present report in addition to our previous lab studies^17^ suggests the therapeutic application of neferine with its differential role in normal and cancer cell line for the combinatorial therapy in cancer treatment with DOX, minimizing the major cardiac side effects. Figure 7 depicts the mechanistic action of neferine on DOX-mediated toxicity in H9c2 cells.

## Materials and Methods

### Chemicals, reagents and antibodies

Doxorubicin, neferine, DCF-DA, FURA 2/AM, JC-1, DAPI, rotenone, lysis buffer (CelLytic™), U0126 ERK1/2 and SB203580 p38 inhibitor were purchased from Sigma (Bangalore, India). Dulbecco’s Modified Eagle’s Medium (DMEM), Fetal Bovine Serum (FBS) and antibiotic solutions were obtained from HiMedia Laboratories (Mumbai, India). DiOC6 (3,3′-dihexyloxacarbocyanine iodide) and CCCP [carbonyl cyanide 4-(trifluoromethoxy) phenylhydrazone] were obtained from Calbiochem, (San Diego, CA, USA). PVDF membranes were obtained from Whatman (Clifton, NJ, USA). Protein marker (PageRuler™) and all other chemical used for SDS-PAGE were obtained from Thermo Scientific (Bangalore, India). Primary monoclonal antibodies for p22phox (sc-20781), p47phox (sc-14015), gp91phox/Nox2 (sc-5827), p-ERK1/2 (sc-7383), p-p38 (sc-7973), NfκB (sc-109), COX2 (sc-1745), TNF-α (sc-1350), Bcl-2 (sc-7382), Bax (sc-526), p-Bad (sc-101641), cytochrome C (sc-13560), caspase-9 (sc-8355), β-actin (sc-47778), α-tubulin (sc-5286), cyclin D1 (sc-246), GAPDH (sc-25778), HDAC1 (sc-7872) and HRP conjugated secondary antibodies raised against mouse, rabbit and goat were purchased from Santa Cruz Biotechnology (Santa Cruz, CA, USA). Antibody against caspase-3 (#9665 s) was purchased from Cell Signalling Technology (Danver, MA 01923, USA).

### Cell culture

Rat cardiomyoblast cells, H9c2 was purchased from National Centre for Cell Science (NCCS), Pune, India. Cells were cultured in DMEM with 10% FBS containing antibiotics (penicillin and streptomycin) in 75 cm^2^ flask. Cell culture flask was maintained at 37 °C in a humidified CO_2_ incubator (Nuaire make) with 5% CO_2_ supply. Cells were sub-cultured at 80% confluency and media was changed once in two days. All the experiments were performed within 20–40 passages in order to confirm cell population, uniformity and reproducibility.

### Cell viability determination by MTT assay

3-(4,5-dimethylthiazol-2-yl)- 2,5-diphenyltetrazolium bromide (MTT) assay was used to determine the cell viability^56^. H9c2 cells were seeded in 96 well plates with cell population of 1 × 10^4^ cells/well and allowed for attachment overnight at 37 °C in CO_2_ incubator. Neferine dissolved in 50% ethanol (v/v) (final working concentration not exceeding 0.01%) and DOX dissolved in deionized water were used. H9c2 cells were treated with neferine (1, 2.5, 5, 7.5, 10, 15 and 20 µM) for 24 h and DOX (0.1, 0.15, 0.25, 0.5, 0.75, 1, 5, 7.5, 10, 15, 20, 25 and 50 µM) for 24 h. After treatment period, cells were briefly washed with PBS and 20 µl of MTT (5 mg/ml) was added to each well and incubated for 4 h at 37 °C in CO_2_ incubator. The formazan crystals formed were dissolved by adding 200 µl of DMSO and mixed well. The optical density (OD) of each sample was measured at 570 nm using Epoch™ Microplate Spectrophotometer (BioTek, Winooski, VT, USA). Results were expressed as % of cell viability.

### ROS estimation

ROS generation in the H9c2 cells were quantified using DCF-DA fluorescent probe according to Lebel, *et al*.^57^. 1 × 10^5^ cells were seeded in 24 well plates and allowed for attachment overnight. Cells were incubated with 10 µM of DCF-DA for 30 min at 37 °C and exposed to DOX for various time points (0.5 h, 1 h, 2 h, 3 h, 4 h, 5 h and 6 h) for time course experiment. In neferine pre-treatment, the cells were exposed to neferine for 24 h followed by DOX for the peak ROS accumulation. At the end of treatment period, cells were harvested, washed with PBS and lysed using Triton X-100. Finally cells were suspended in PBS and fluorescent intensity was measured at Ex/Em, 480/520 nm using Synergy H1 Multi-Mode Reader (BioTek, Winooski, VT, USA). The accumulation of ROS in H9c2 cells were also analyzed visually by using fluorescence microscope (Olympus, Japan) with the Ex/Em of DCF.

### Measurement of intracellular calcium [Ca^2+^]i

Intracellular calcium in the H9c2 cells was estimated as described earlier^58^. 1 × 10^5^ cells were cultured in 24 well plates and treated with DOX for various time points (0.5 h, 1 h, 2 h, 3 h, 4 h, 5 h and 6 h) to determine the peak accumulation of calcium. In neferine pre-treatment, the cells were exposed to neferine for 24 h followed by DOX at the peak ROS accumulation. At the end of treatment period, cells were washed with buffer A twice for 5 min (5 mM KCl, 2 mM CaCl_2_, 0.5 mM KH_2_PO_4_, 137 mM NaCl, 4 mM NaHCO_3_, and 0.2 mM Na_2_HPO_4_). The cells were incubated with 4 µM of Fura 2/AM for 45 min and fluorescent intensity of the dye was measured at Ex/Em, 340–380/500 nm in Synergy H1 Multi-Mode Reader (BioTek, Winooski, VT, USA).

### Preparation of protein extract and immunoblotting

For analyzing NFκB expression, nuclear extracts were used. NE-PER™ Nuclear and Cytoplasmic Extraction Reagents (Thermo Fisher Scientific Inc.) was used to separate the nuclear and cytoplasmic proteins according to manufacturer’s instruction. For total protein extraction, CelLytic™ (Sigma-Aldrich) lysis buffer was used. Bradford method was used to quantify the extracted protein. Equal amount of protein was separated in 12% gel using SDS-PAGE unit (Bio-Rad Laboratories, Hercules, CA, USA) at 100 V for 2 h. Separated proteins from the gel were blotted to activated PVDF membrane using semi-dry transfer unit (Hoefer Inc., Holliston, MA, USA) at 90 V for 1.5 h. Blots were blocked using 5% non-fat milk powder in TBST for 1 h at room temperature. Then the blots were washed with TBST thrice for 5 min each. After that, blots were incubated with specific primary antibody at 4 °C for overnight. Again blots were washed with TBST thrice for 5 min each and incubated with corresponding HRP-conjugated secondary antibody for 1 h at room temperature. Finally the blots were visualized using ECL substrate and images of the blots were analyzed densitometrically using ImageJ software.

### Measurement of mitochondrial membrane potential (ΔΨm)

Mitochondrial membrane potential (ΔΨm) in different treatment group was estimated using DiOC6^59^. After treatment period, cells were washed with PBS and incubated with 50 nM of DiOC6 for 30 min at 37 °C. CCCP (50 µM) was used as a positive control for inducing mitochondrial depolarization. Cells were washed with PBS once, resuspended in PBS and fluorescent intensity of DiOC6 measured at Ex/Em, 488/500 nm using Synergy H1 Multi-Mode Reader (BioTek, Winooski, VT, USA). The intact and depolarized mitochondria of H9c2 cells were also analyzed visually by JC-1 staining as per manufacturer’s protocol using EVOS fluorescence microscope (Life Technologies, USA) with Ex/Em, 585/590 nm for normal mitochondria and Ex/Em, 514/529 nm for unhealthy mitochondria.

### Cell cycle analysis

1 × 10^6^ cells were seeded in 6 well plates and treated with appropriate dose of neferine and DOX. At the end of treatment period, the cells were harvested, washed with PBS and fixed in 70% ethanol at 4 °C for overnight. Ethanol was removed, cells were washed using PBS and suspended in 500 µl of propidium iodide (50 µg/ml) containing 0.1% Triton X-100, 0.1% sodium citrate, and 20 µg/ml of RNase. Finally the samples were analyzed in BD 290 flow cytometer (J. Trotter, San Diego, CA, USA) using Win MDI flow cytometric software (BD Biosciences, San Jose, CA, USA) to analyze cell cycle distribution.

### Terminal Deoxynucleotidyl Transferase-mediated Nick-End Labeling (TUNEL) assay

TUNEL assay was performed using *In Situ* Cell Death Detection Kit (Roche) according to manufacturer’s instruction. Briefly at the end of treatment, the cells were washed with PBS and fixed using 4% paraformaldehyde for 30 min. Permeation solution (0.1% Triton X-100 in 0.1% sodium citrate) was added to the cells for 10 min. After a gentle wash with PBS, cells were incubated with TUNEL reagent for 1 h. Finally cells were stained with DAPI for 5 min and observed under fluorescent microscope (Olympus, Japan).

### Scanning Electron Microscopy

5 × 10^4^ cells were cultured in cover slip for overnight and treated with neferine and DOX. After treatment, cells were fixed using 2% paraformaldehyde and 2.5% glutaraldehyde in 0.1 M sodium cacodylate buffer (pH 7.4) as primary fixative for 1 h. Cells were washed with sodium cacodylate buffer to remove excess fixative. Secondary fixative, 1% OsO4 in 0.1 M cacodylate buffer (pH 7.4) was added to the cells and incubated for 1 h. Cells were then dehydrated using different graded ethanol (30%, 60%, 90% and 100%) for 30 min each. The samples were kept dry in a vacuum desiccator until microscopic analysis and visualized under scanning electron microscope (FEI QUANTA 200, Germany).

### Statistical Analysis

Results were expressed as mean ± s.d. One-way ANOVA followed by Tukey’s multiple comparison test was used for all the experiments. SPSS software Version 17 (SPSS, Chicago, IL) was used for performing statistical analysis.

Note: Please see Supplementary Data S1 for uncropped western blots.

## Electronic supplementary material

Supplementary Data S1
