# Supplementary material for: Neferine ameliorates cardiomyoblast apoptosis induced by doxorubicin: possible role in modulating NADPH oxidase/ROS-mediated NFκB redox signaling cascade
Source: Sci Rep. 2017 Sep 25;7:12283. doi: 10.1038/s41598-017-12060-9 (PMC5612945; doi:10.1038/s41598-017-12060-9)
Supplement: Supplementary file 1 — Supplementary Data S1 [file 41598_2017_12060_MOESM1_ESM.pdf]

Neferine ameliorates cardiomyoblast apoptosis induced by doxorubicin: possible role in modulating NADPH oxidase/ROS-mediated NFκB redox signaling cascade

Lohanathan Bharathi Priya[1], Rathinasamy Baskaran[2], Chih-Yang Huang[3,4,5], Viswanadha Vijaya Padma[1,3,5,*]

Supplementary Data S1: Uncropped western blots

1D

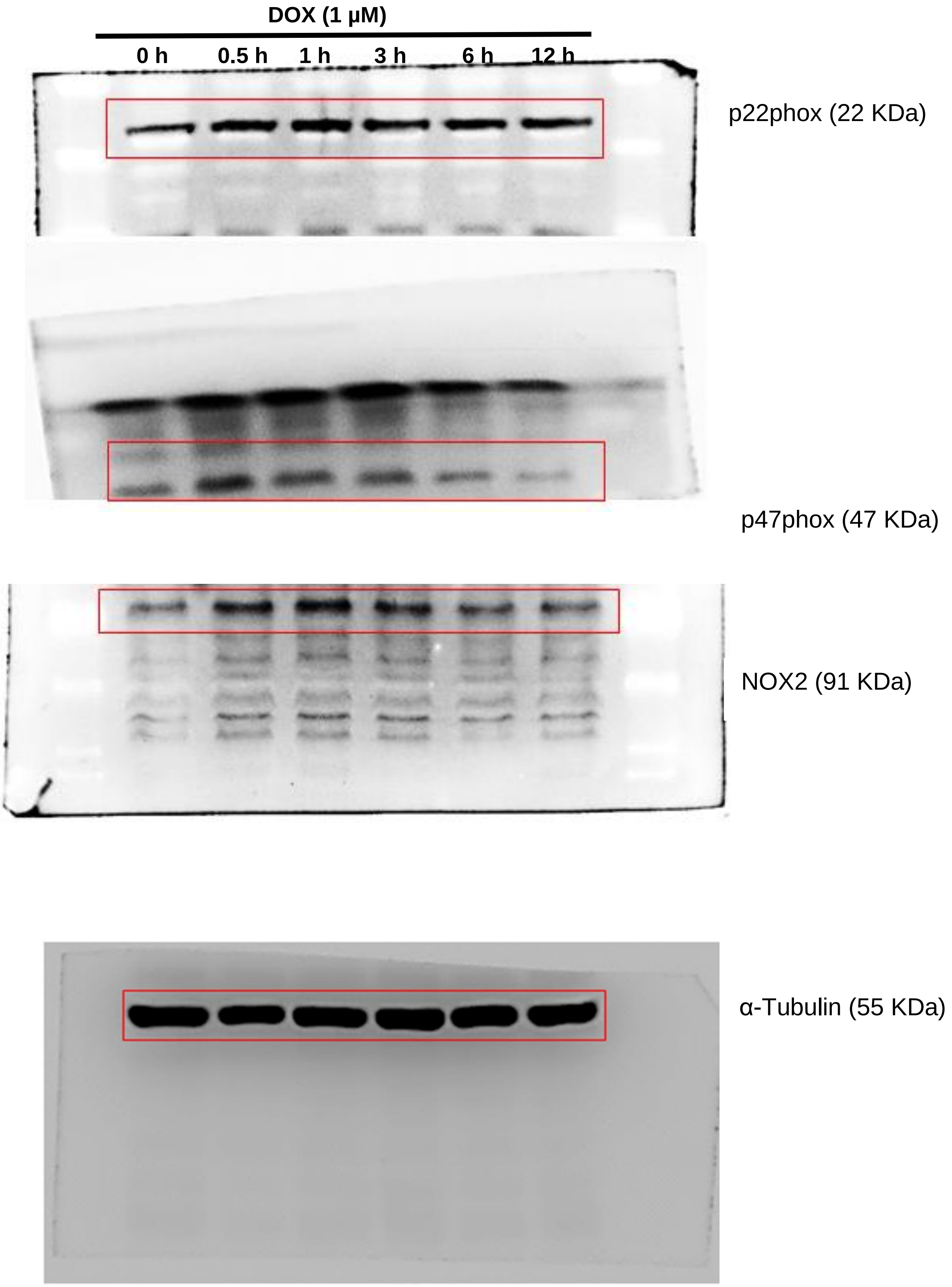

1E

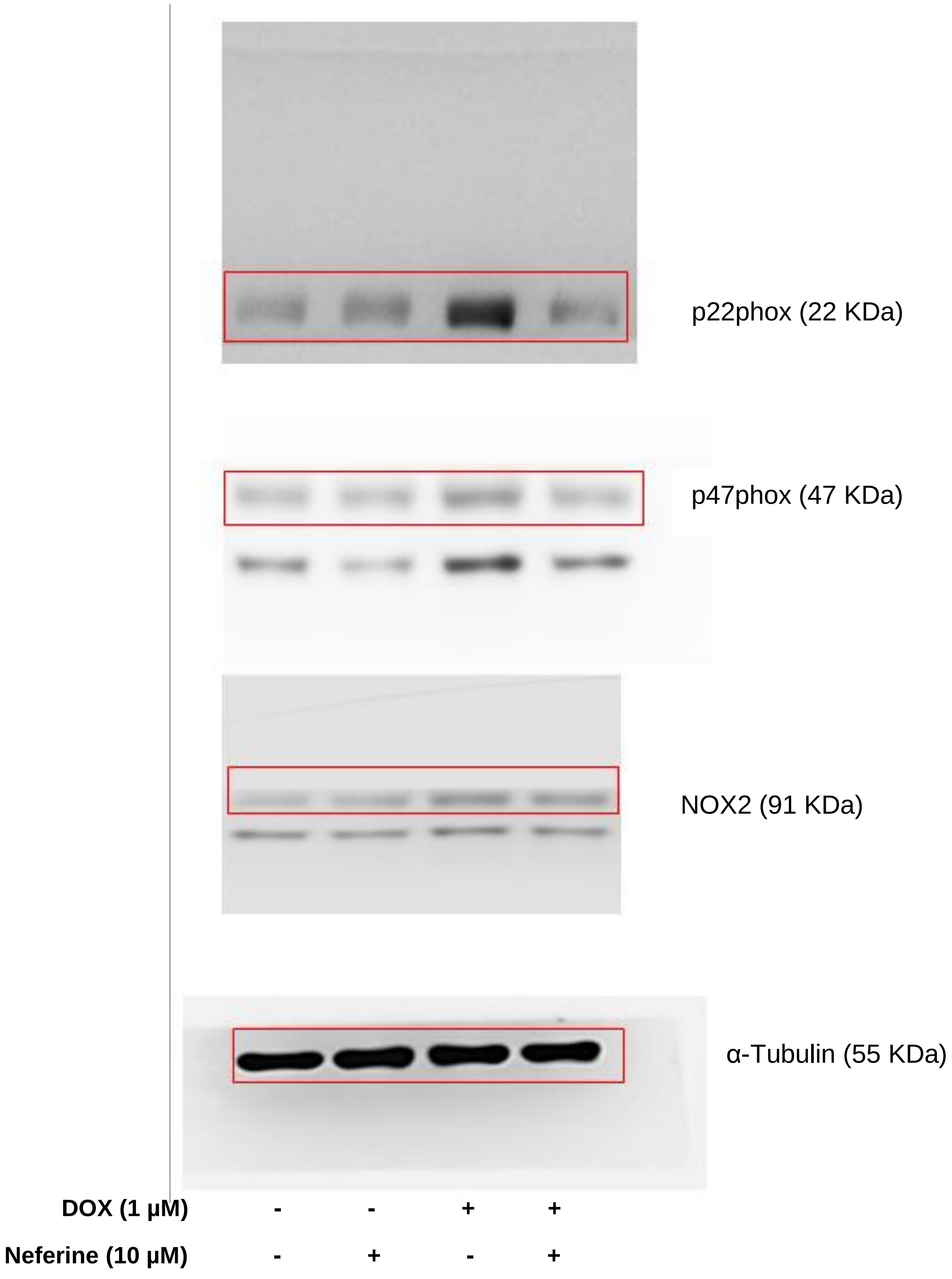

p22phox (22 KDa)
p47phox (47 KDa)
NOX2 (91 KDa)
α-Tubulin (55 KDa)
DOX (1 μM) - - + +
Neferine (10 μM) - + - +

2D

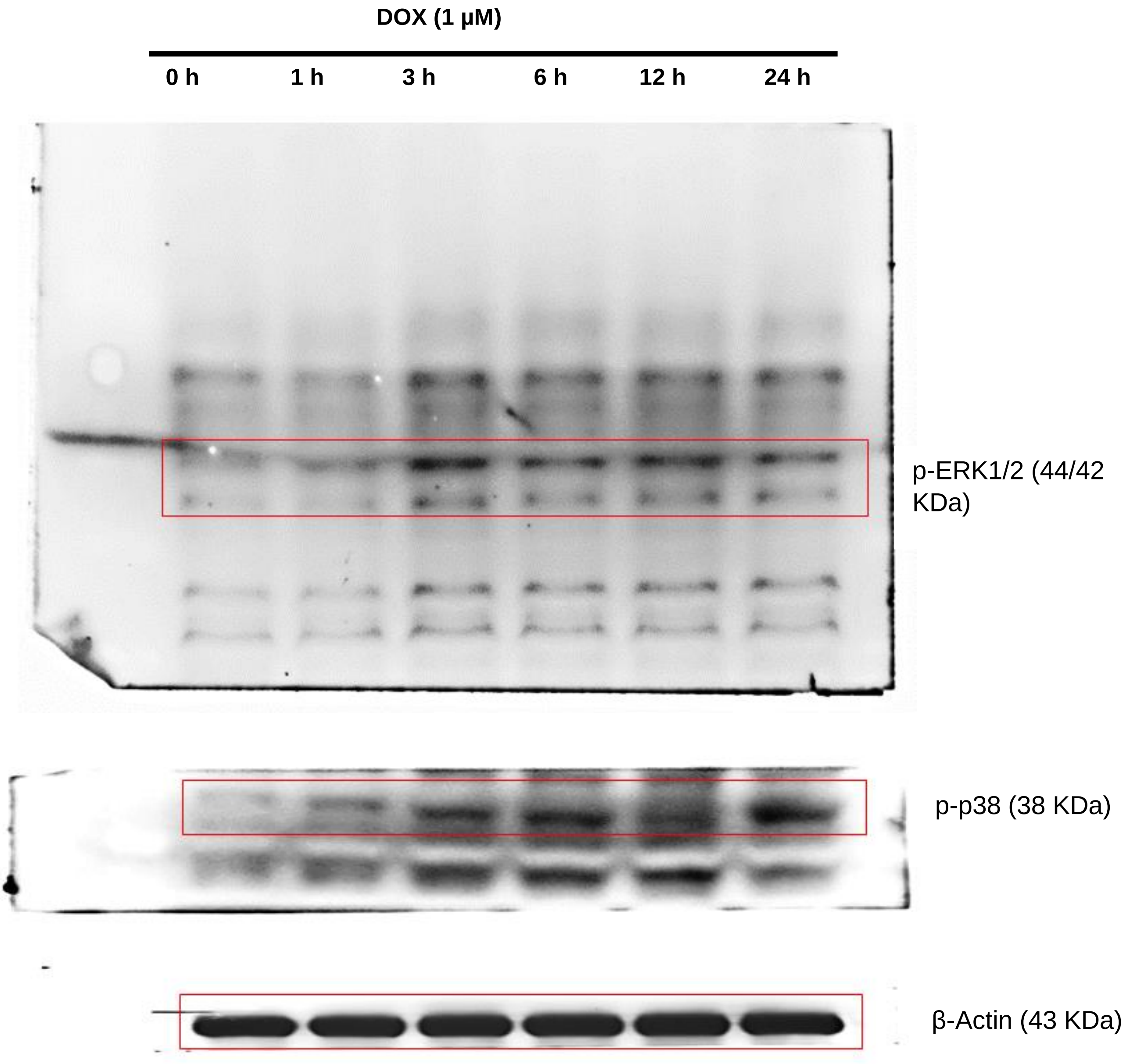
DOX (1 μM)
0 h
1 h
3 h
6 h
12 h
24 h
p-ERK1/2 (44/42 KDa)
p-p38 (38 KDa)
β-Actin (43 KDa)

2E

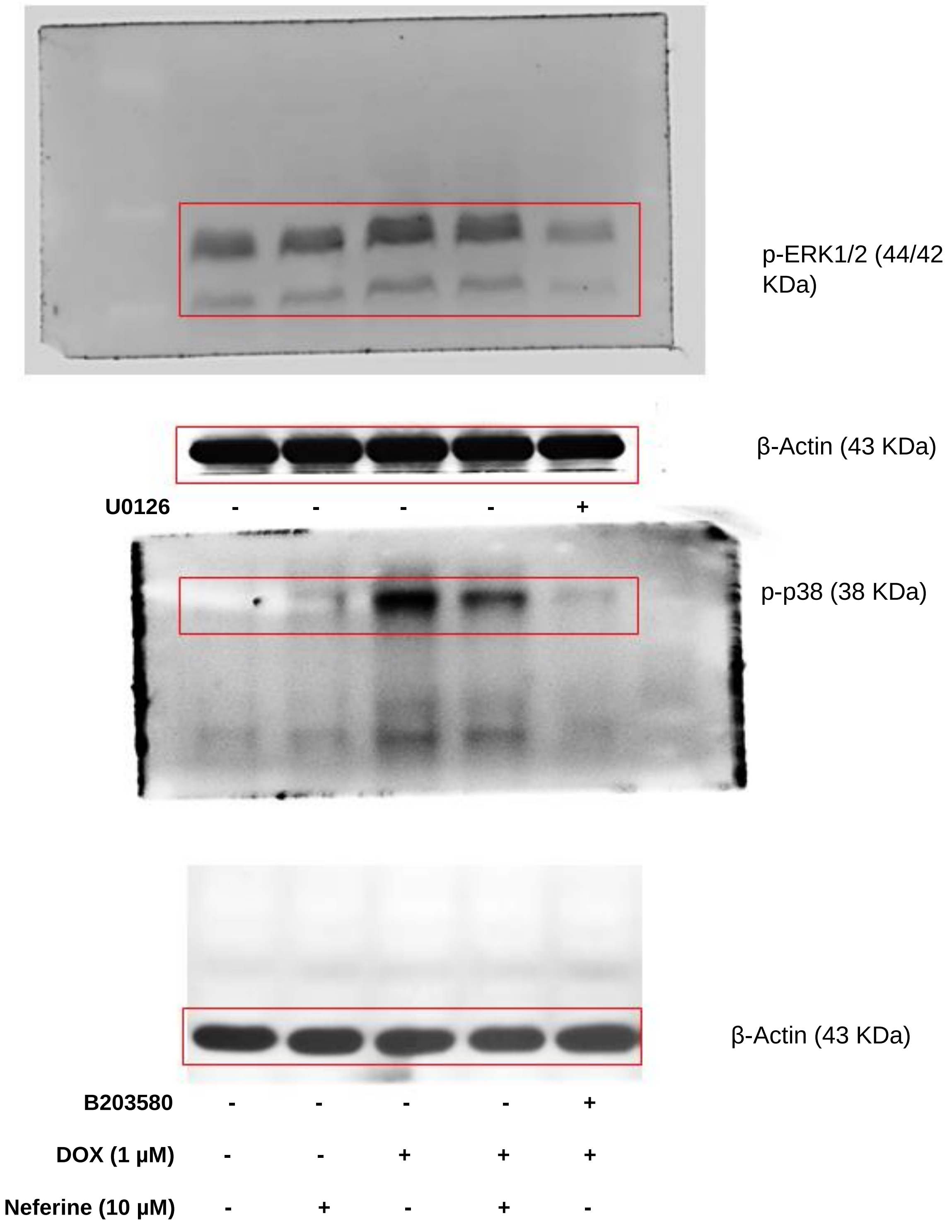
p-ERK1/2 (44/42 KDa)
β-Actin (43 KDa)
U0126 - - - - +
p-p38 (38 KDa)
β-Actin (43 KDa)
B203580 - - - - +
DOX (1 µM) - - + + +
Neferine (10 µM) - + - + -

3A

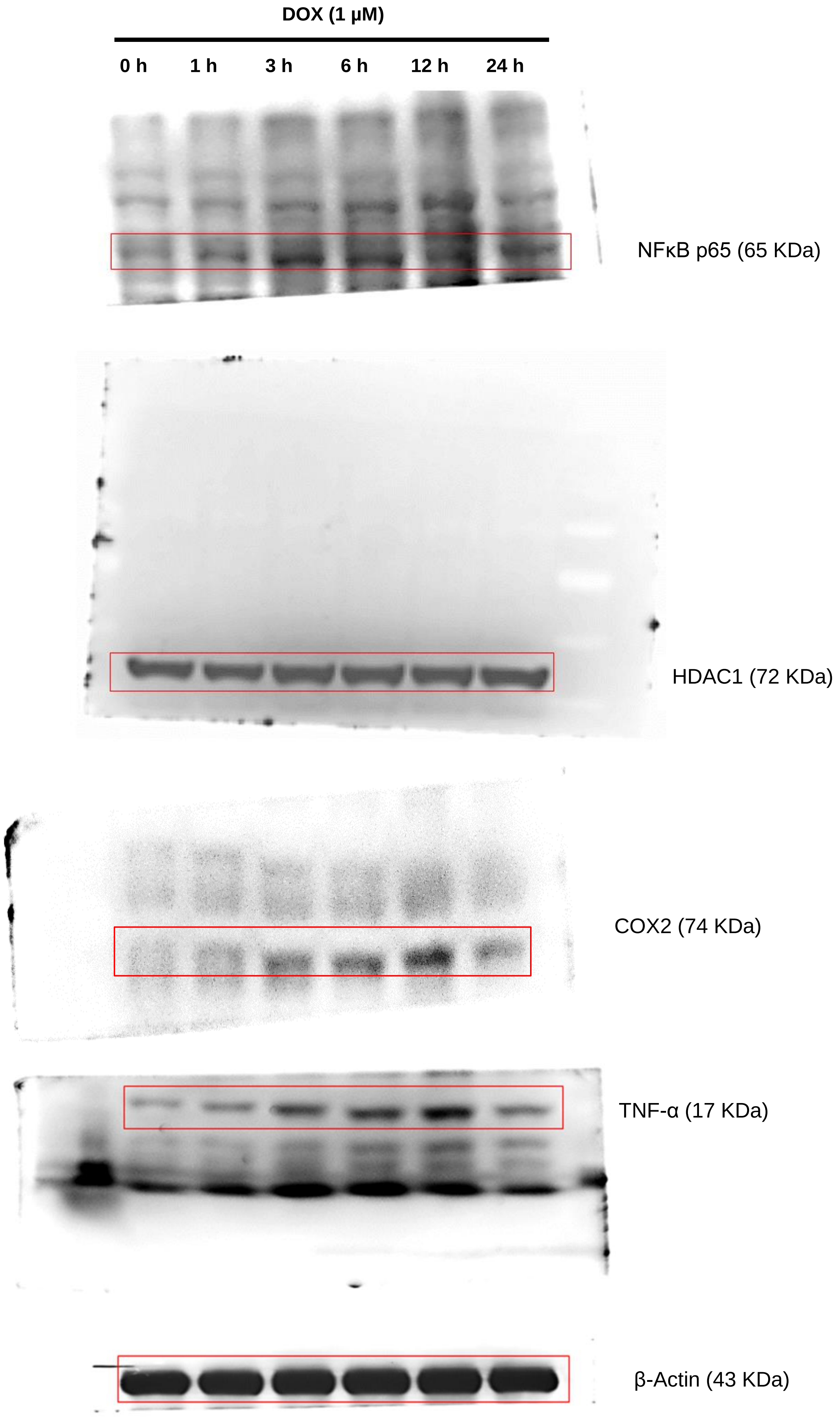
DOX (1 μM)
0 h
1 h
3 h
6 h
12 h
24 h
NFκB p65 (65 KDa)
HDAC1 (72 KDa)
COX2 (74 KDa)
TNF-α (17 KDa)
β-Actin (43 KDa)

3B

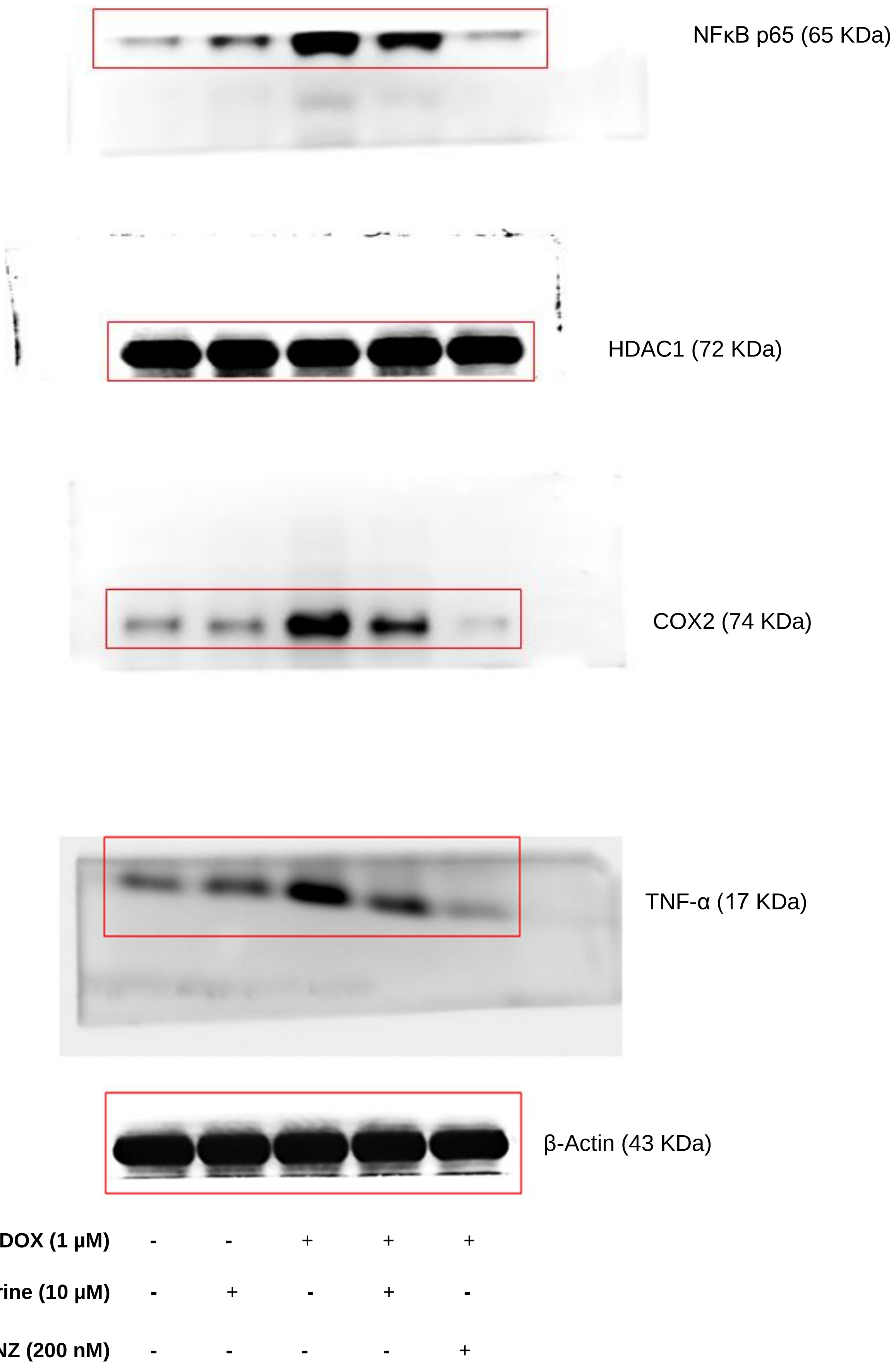
NFκB p65 (65 KDa)
HDAC1 (72 KDa)
COX2 (74 KDa)
TNF-α (17 KDa)
β-Actin (43 KDa)
DOX (1 μM) - - + + +
Neferine (10 μM) - + - + -
QNZ (200 nM) - - - - +

5D

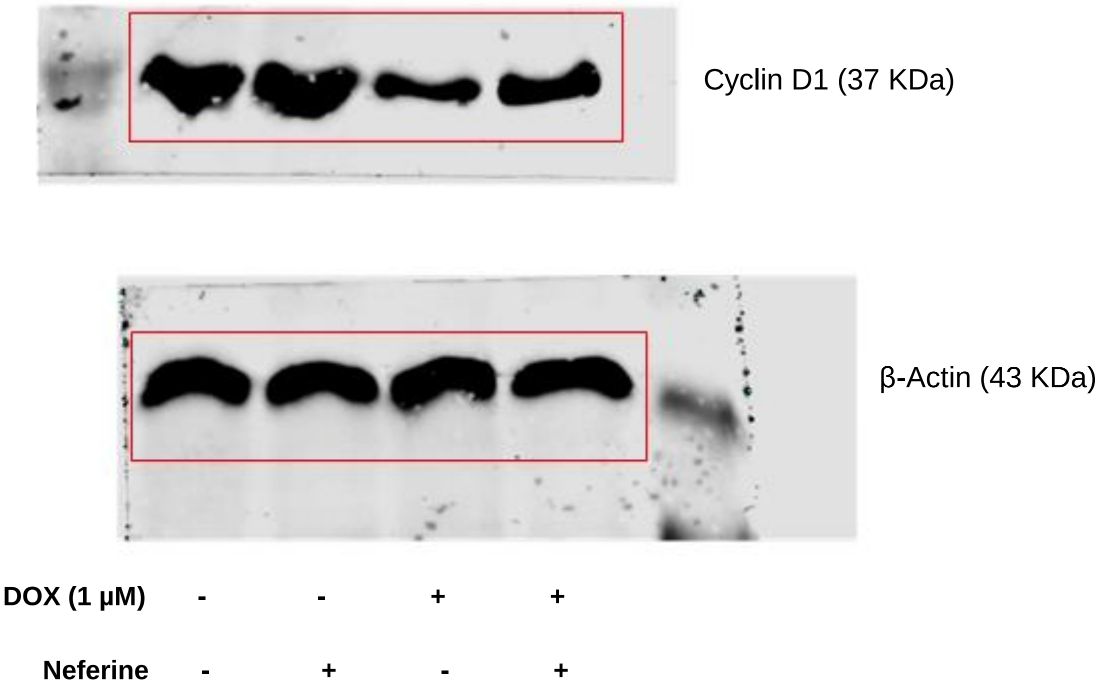

6C

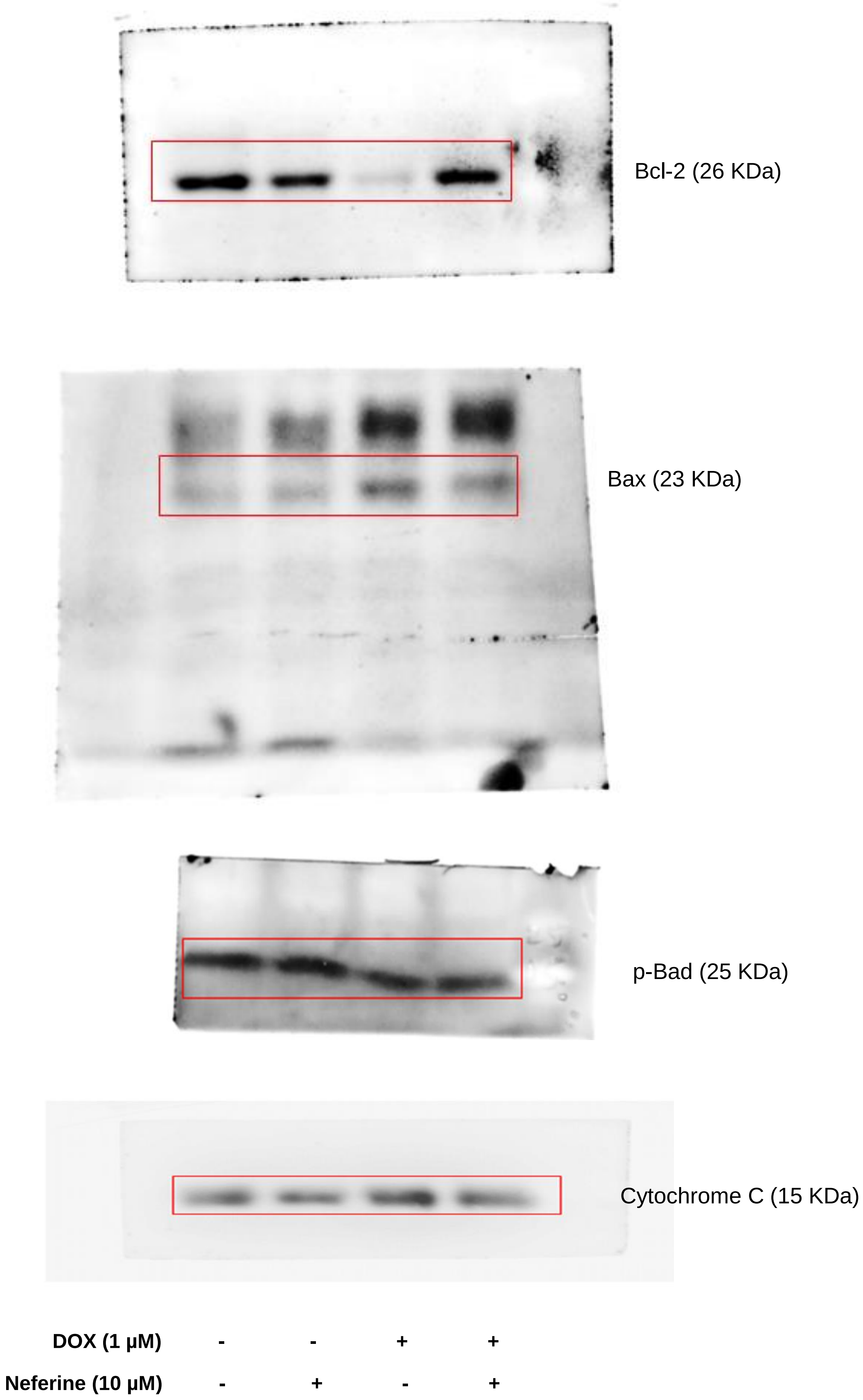
Bcl-2 (26 KDa)
Bax (23 KDa)
p-Bad (25 KDa)
Cytochrome C (15 KDa)
DOX (1 µM) - - + +
Neferine (10 µM) - + - +

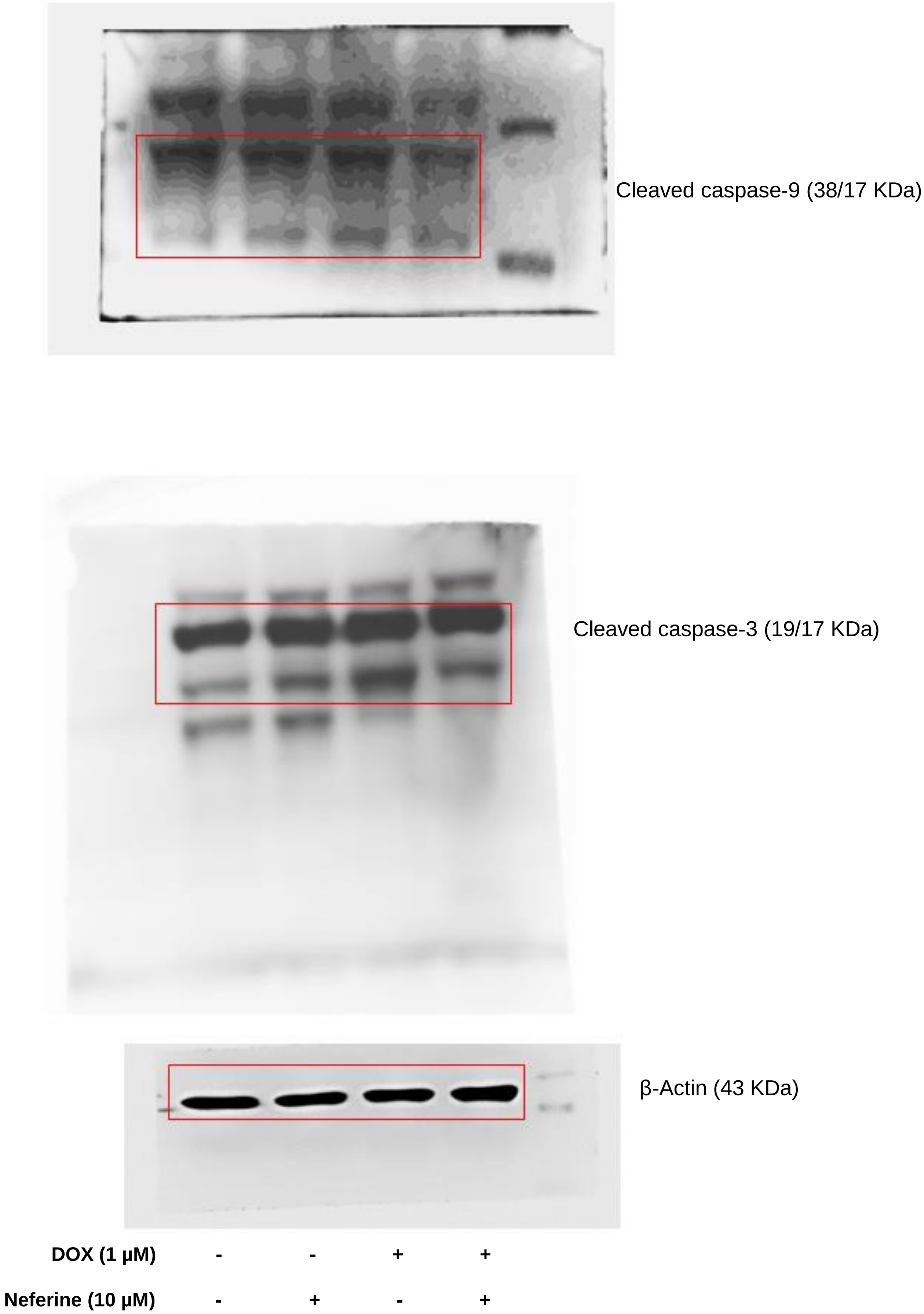
Cleaved caspase-9 (38/17 KDa)
Cleaved caspase-3 (19/17 KDa)
β-Actin (43 KDa)
DOX (1 μM) - - + +
Neferine (10 μM) - + - +
